# Peer-teaching at the University of Rwanda - a qualitative study based on self-determination theory

**DOI:** 10.1186/s12909-020-02142-0

**Published:** 2020-07-20

**Authors:** Alexis Nshimiyimana, Peter Thomas Cartledge

**Affiliations:** 1 College of Medicine and Health Sciences, University of Rwanda, Kigali, Rwanda; 2 Department of Paediatrics, University Teaching Hospital of Kigali (CHUK), Kigali, Rwanda; 3 Rwanda Human Resources for Health (HRH) Program, Ministry of Health, Kigali, Rwanda; 4 Department of Emergency Medicine, Yale University, New Haven, Connecticut USA; 5 Department of Paediatrics, School of Paediatrics, University of Leeds, Leeds, UK

**Keywords:** Peer-teaching, Peer-learning, Students, medical, Self-determination theory, Education, medical, Pediatrics, Developing countries, Rwanda

## Abstract

**Background:**

Peer-teaching is an educational format in which one student teaches one, or more, fellow students. Self-determination theory suggests that intrinsic motivation increases with the enhancement of autonomy, competence and relatedness.

**Aims:**

This qualitative study sought to explore and better understand the lived experiences, attitudes and perceptions of medical students as peer-teachers at the University of Rwanda when participating in a peer-learning intervention in the pediatric department.

**Methods:**

Students participated in a 3-h peer-taught symposium, supervised by a pediatric specialist or resident. Students worked in small groups to deliver a short didactic presentation related to acute illness in children. The symposium prepared the students for simulation-based teaching activities. In-depth, semi-structured, interviews were then employed to explore the students’ experiences of the peer-teaching symposium. We specifically aimed to scaffold the analysis of these experiences on the themes of autonomy, competence and relatedness.

**Results:**

Saturation was achieved after interviews with ten students. Students described developing their own autonomous learning strategies, but despite developing this autonomy had a desire for support in the delivery of the sessions. Competence was developed through enhanced learning of the material, developing teaching skills and confidence in public speaking. Students valued the different aspects of relatedness that developed through preparing and delivering the peer-teaching. Several other themes were identified during the interviews, which are not described here, namely; i. Satisfaction with peer-teaching; ii. Peer-teaching as a concept; iii. Practical issues related to the peer-teaching session, and iv. Teaching style from faculty.

**Conclusions:**

This is the first study to assess peer-learning activities in Rwanda. It has used qualitative methods to deeply explore the lived experiences, attitudes and perceptions of medical students. The peer-teaching strategy used here demonstrates the potential to enhance intrinsic motivation while increasing knowledge acquisition and teaching skills. We postulate that students in resource-limited settings, similar to Rwanda, would benefit from peer-teaching activities, and in doing so could enhance their intrinsic motivation.

## Background

### Peer-teaching in medical education

Peer-teaching is defined as “People of similar social groupings who are not professional teachers helping each other to learn and learning themselves by teaching” [1, 2]. In resource-limited settings, beyond its educational benefits, peer-teaching and near-peer teaching may have practical benefits, such as reducing the workload on academic faculty where faculty to student ratios are low [3, 4]. Students who teach (peer-teachers, also known as peer-tutors) also benefit, stimulating high-level processing of the information during the preparation and delivery of the teaching, collaborative and independent research as well as feedback on their teaching [1, 5–7]. Peer-teaching has the potential to improve the student’s acquisition of knowledge and skills that can be applied in future clinical situations [8–10]. It increases students’ confidence in clinical practice, participation, leadership, learning opportunities, and can increase student-teacher satisfaction [2]. Subjective measures of peer-teaching support social and cognitive congruence hypotheses and demonstrate that peer-teaching is positively received by learners [4].

### Impact of implementing peer-teaching

A 2011 systematic review of studies from developed countries, suggested peer-teaching to have both academic and professional benefits on student-teacher learning outcomes [2]. A further systematic review in 2016 specifically compared controlled studies of peer-teaching with faculty delivered teaching in terms of knowledge and skill acquisition, and found that students taught by peers did not have significantly different outcomes to those taught by faculty and that peer-teaching should be supported [4]. Negative aspects of peer-teaching include reduced student learning if personalities or learning styles are not compatible, students spending less individualized time with the clinical instructor and anxiety for the peer-teacher [11–14]. These challenges require careful consideration and may warrant bespoke responses in order to optimize the learning experience for students and a consideration of whether a more suitable educational activity is available. Documented evidence of peer-teaching and Peer Assisted Learning (PAL) activities remains uncommon in low-income countries, Rwanda included. Our own experience is that more traditional, didactic, forms of teaching are more prevalent in this setting.

### Motivation

Humans can be engaged and proactive, or alternatively, passive and disengaged. The motivation for particular activities is largely a function of the social conditions in which humans develop and function [15, 16]. Motivation can also be affected by age, gender, ethnicity, and socio-economic status [17, 18]. Modifiable factors can have the potential to affect the motivation of students, traditionally classified as extrinsic or intrinsic motivators. Extrinsic motivation (EM) refers to the performance of an activity driven by external rewards, such as status, money or passing grades [15–17]. Though extrinsic motivators may be effective in the short-term, they may also be detrimental in the long-term as it has been shown that they undermine intrinsic motivation [15, 19]. A person is intrinsically motivated (IM) if they perform an activity for no apparent reward except the reward of the activity itself [15, 17, 20]. In the AMEE guide on Self Determination Theory (SDT), Ten Cate states that “High IM, e.g. learning out of interest, curiosity or enjoyment, and autonomous forms of self-regulation are associated with better learning, better conceptual understanding, better academic performance and achievement and higher levels of well-being than high extrinsic motivation” [3]. Within our own setting, most Rwandan medical students enter medical school directly after having completed high-school. In Rwandan culture and society, medicine is considered as a career choice for school students who have performed exceptionally well academically at high-school. Our personal experience, though not measured, is that the motivation to pursue medical studies often comes from external sources, such as parents, schools and communities, rather than the students themselves.

### Self-determination theory

SDT proposes that human beings have a natural tendency to develop towards learning, growth and intellectual challenge, thus self-determination [17, 21]. Within SDT, IM is always associated with the fulfilment of the need for three key factors, namely; i. Autonomy, ii. The need for competence and iii. The need for relatedness [3, 17]. The fulfilment of these three basic psychological needs makes a person intrinsically motivated for a particular activity [17]. SDT puts forth autonomous motivation as a positive and therefore desired type of motivation leading to reduced superficial information processing, and enhanced well-being or adjustment along with the other positive associations of IM [17, 22–26]. In light of SDT, much of the energy invested in educational activities by medical educators and curriculum developers could be better channeled to finding ways to stimulate autonomous forms of motivation [1, 3]. Motivation can be quantitatively measured using tools, based on SDT, such as the Strength of Motivation for Medical School (SMMS) questionnaire, however these tools have not been formally validated in our setting and they do not give a rich and detailed understanding of the experiences, attitudes and perceptions of students [27].

### Study aims

Peer-teaching is a relatively novel medical education strategy, especially within the Rwandan medical curriculum. There is therefore little known on how this strategy would be experienced by this group of students. This qualitative study sought to explore and better understand the lived experiences, attitudes and perceptions of medical students as peer-teachers at the University of Rwanda when participating in a Peer-Assisted Learning intervention in the pediatric department. This can be described by the specific research question of; what are the experiences, attitudes and perceptions of Rwandan medical students as peer-teachers and peer-learners? We sought to explore and describe these lived experiences scaffolded within the three categories of autonomy, competence and relatedness as described in Self-Determination theory.

## Methods

### Study design

A qualitative study was undertaken. This study has been reported in accordance with the COREQ and SRQR checklists for qualitative research and the GREET checklist for medical education research [28–30].

### Why a qualitative approach was taken?

We aimed to gain a rich and detailed understanding of the lived experiences, attitudes and perceptions of students, and for the results to emerge from the data. Therefore, limiting the enquiry to fixed quantitative measures was felt overly restrictive.

### Qualitative research approach and guiding theory

A phenomenological approach was used based on the guiding theory of SDT.

### Setting

Rwanda is a small, landlocked country with a population of 12 million. The University of Rwanda (UR) is a public, single, multi-campus institution established in 2013 from the merger of the nation’s seven public Higher Learning Institutions into a single public university with six self-governing colleges of which the College of Medicine and Health Sciences (CMHS) is a part [31]. The school of medicine intake is approximately 80% male. The study was conducted at the University Teaching Hospital of Kigali (CHUK), a public, tertiary hospital located in Kigali City, the capital of Rwanda. CHUK hospital is a major teaching site for UR. Year-five students currently undertake pediatric rotations at CHUK hospital.

### Learning objectives of the educational intervention

On graduation, medical students in Rwanda can often be placed to provide clinical care in isolated rural settings, immediately responsible for large numbers of patients, including pediatric care. The objective of the symposium was to give students the theoretical knowledge they required to provide this care and also to prepare them to participate in planned simulation-based learning activities to develop competencies. The teaching method used to meet the symposium objectives was peer-teaching.

### Intervention

Students attended and taught a three-hour symposium, supervised by a pediatric specialist or resident. The symposium was on the assessment and management of acutely unwell children (ABCDE). The materials were based upon the “Pediatric Recognition and Responding to Acute Patient Illness and Deterioration” (pRRAPID) course from the University of Leeds (UoL), UK, with locally relevant adaptions [32]. The symposium comprised of seven 15-min, didactic presentations. Groups of 2–3 students were each assigned one presentation topic to deliver to their peers as “peer-teachers”. As we wanted to ensure the symposium kept to time and to ensure the content of the sessions were factually correct the groups of students were provided with pre-prepared PowerPoint slides to guide their presentation, along with a reference e-book/App relevant to their talks, available for free, online [33, 34]. Students were given a written explanation of the symposium and soft (electronic) copies of the Power-Point slides in advance of the session in order to give them time to prepare with their group. Peer-teachers and their students were all from the same class at the same institution (UR). The symposiums took place in a classroom, suitably sized for the number of students. Students were not assessed summatively, rather being given only formative feedback on their presentation from the supervisor. The peer-teaching symposium and PowerPoint materials had already been piloted and delivered in the United Kingdom (UK) by one of the authors (PC). Locally relevant adaptations for the Rwandan students and context were made. No changes to the planned teaching occurred after initiating the educational intervention. Symposiums were held as groups of students rotated through the pediatric department. This symposium was then followed by a series of simulation-based learning (SBL) sessions which were not peer-taught and were led by experts. The SBL activities have not been assessed in this research project.

### Qualitative design

In-depth, semi-structured interviews were used to explore the experiences of undergraduate (UG) medical students regarding peer-teaching and the meanings they attribute to it. The interviewer encouraged participants to discuss issues pertinent to the research questions by asking open-ended questions in one-to-one interviews.

### Participants

Undergraduate year-five medical students in the penultimate year of their six-year programme. Batches of students rotate into the pediatric department every 4 weeks, and the Peer-Assisted Learning symposium was implemented with each group of 18–25 students.

### Selection of study participants

#### Inclusion criteria

Students who had undertaken the pRRAPID peer-taught symposium within the last 6 months.

### Sampling

Purposive sampling was employed, explicitly selecting interviewees using “typical case sampling” who were likely to generate appropriate and useful data, and including sufficient participants to answer the research questions and achieve saturation [35, 36].

#### Enrolment

An invitation to take part was sent to all eligible year-five students via the class representative using e-mail and the class WhatsApp group. Participants volunteered to take part in the study. The class representative provided a list of students who had volunteered to take part. Eligible students were enrolled in the order provided by the class-representative. Eligible participants were informed of the proposed interview date, time, and meeting place 2 days before the interview. If the volunteer was not available at the set time then the next volunteer on the list was enrolled.

### Procedures after enrollment

Potential participants were provided with a study information leaflet. Once informed, written consent was gained. We then undertook a one-on-one semi-structured interview. All interviews were conducted in English. No field notes were taken during the interview. The interviews were recorded digitally using a password-protected smartphone. We conducted a single interview with each participant, with no repeat interviews.

### Data collection tool (interview guide)

Questions were explicitly designed for use in this study and were not pre-validated (Supplementary File 1). The questionnaire was piloted using 1 year-five student in order to test the understanding of the questions and to predict the length of the interview. Appropriate modifications were made after piloting. The questionnaire acted as a framework to guide the conversations. During interviews, the interviewer re-worded, re-ordered or clarified the questions in the interview guide to fully investigate topics introduced by the respondent.

### Setting & methods of the interview

To ensure participants were comfortable enough to explore their experiences, perceptions and reactions, the interviews took place in a quiet room at CHUK hospital without interruptions or any other persons present.

### Incentives

Participating as an interviewee was voluntary, and non-participation did not academically affect non-participants. Students who participated were offered 2000Rwfr (equivalent to $2.40) of mobile phone credit for participating in the study. This was not a payment and rather a proportional reimbursement for the time and public transport to the site for the interview (approximately 1 h).

### Confidentiality

The interview recordings and transcripts were kept, anonymously, in a password-protected laptop.

### Risk to subjects

Emotional and social risks were assessed to be minimal, and participants were not obliged to take part. Students were reminded that the interviews were recorded and that political comments or derogatory comments regarding people would result in the ending of the interview. In this context, political comments were those defined as those regarding the government of Rwanda or about the policies of the University of Rwanda. Derogatory comments about people were defined as any comments that were discriminatory or pejorative regarding any person and not relevant to educational activity. No physical, legal and/or financial risks were identified with this study.

### Interviewer and researcher characteristics, credentials, occupation, experience and training

The Principal Investigator (PI, AN) conducted the interviews. He is a male, Rwandan, year-five undergraduate medical student at the UR who is fluent, competent and trained in the English language. The PI is a member of the same medical school class as the participants. Participants were aware that the study was a requirement for the graduation of the PI from his medical degree. The PI had not previously undertaken qualitative research and was therefore supported by a faculty member (PC) experienced in qualitative research. PC supervised and co-authored the work and is a male medical doctor trained in the United Kingdom, and at the time of the study was an associate professor in pediatrics at Yale University, USA, working on the Human Resources to Health Programme in Rwanda [37, 38]. PC has received training in qualitative methods during his Masters in Child Health and has contributed to previous qualitative research in Rwanda (Yale) and the United Kingdom (UoL). PC had no prior relationship to participants, except for being a member of faculty in the pediatric department. PC was a member of the UoL team who developed the pRRAPID package, working with students in the UK. He also modified the symposium for use in Rwanda. The reseachers ideas, experiences and preconceptions about peer-teaching and PAL, under study here, might influence the interpretation of the interview data.

### Transcription

The PI performed transcription. Transcripts were not returned to the participants for review, comment or amendment. No translation was required or undertaken. Initial transcription was completed after the first six interviews, along with coding. The final interviews were transcribed once saturation was completed.

### Approach to analysis

An integrated approach was taken (i.e. both inductive and deductive). No specific hypothesis was formed prior to the research. The analysis was partially inductive in nature, in that, the aim of the study was to simply describe the lived experiences of the students in this setting. The analysis, was also, somewhat deductive, in that the coding was scaffolded within the three factors of SDT. This decision to scaffold the analysis on the factors of SDT was pre-determined at the point of writing the study proposal. However, the study was not fully deductive, in that we did not aim to verify SDT as a theory or answer a specific hypothesis related to SDT. The codes were not pre-determined and emerged from analysis of the transcripts.

### Saturation

Saturation was defined as being when the information from the interviews produced “little or no change to the codebook” (inductive thematic analysis) [35, 39, 40].

### Coding of the data

Interviews were transcribed in Microsoft Word, followed by coding in Microsoft Excel [41]. We specifically aimed to scaffold the reported experiences, and therefore the codes, on the themes of autonomy, competence and relatedness. The coding was iterative, and de novo, line-by-line, within the transcripts. A code was pre-defined as a label; a name that most exactly describes what that particular unit of text is about [42]. The final code-tree is provided as a supplementary file (See supplementary file 2). The principal investigator transcribed the data and familiarised with it prior to starting coding. Transcription and coding was performed by the PI after the first six interviews and then completed with the next block of four interviews. The coding was then cross-checked by the second author (PC) employing the full block of ten interviews. During this process, the second author reviewed the codes that had already been applied to units of text, and made amendments to which codes were applied to units of text. Any differences in coding were resolved to mutual satisfaction.

### Thematic analysis

We employed conventional content analysis, aiming “to provide knowledge and understanding of the phenomenon under study” [43]. Initial analysis of the data was undertaken after the first six interviews before undertaking the final block of four interviews. After the first six interviews the two researchers met to discuss the initial coding tree and the themes developing within the analysis. Consensus was gained on any initial coding or analysis issues. After six-interviews, saturation was discussed in the context of the codes and analysis produced during the first block of interviews. No changes to the data-collection tool were made at this point. After the next block of four interviews it was concluded that saturation was achieved because no new codes emerged. Any differences in the thematic analysis was resolved to mutual satisfaction.

### Potential biases

Eligible participants volunteered to enroll in the study. Subconscious or intentional selection of participants who are more vocally dynamic or those who possess better English language skills may have biased the results. These participants are potentially more likely to be naturally comfortable with PAL and may, therefore, give a biased viewpoint of peer-teaching in their interviews. As we relied on participants to volunteer there were no mitigating steps were taken to minimize this potential bias. Participants were informed that any political comments (about the government of Rwanda or UR) or derogatory comments (pejoratives about persons) would results in the interview being discontinued. Though this was to protect participants, whose interviews were being recorded, it may have introduced bias, in that participants may not have felt able to express themselves fully, especially their negative feelings towards the PAL activity. The interviewer (AN) was a member of the same class of students; this may have introduced response bias. However, this relationship may also have been a benefit, with the interviewer having prolonged involvement with participants, who therefore could have felt more comfortable to share their views with a colleague/peer whom they respect and trust. In particular, the data may be biased by social desirability bias. Though we felt that the interviewer being a peer, rather than a faculty member, will have minimized this. No formal steps were taken to measure or alter these biases. The coding was initially undertaken by the principal investigator (AN) and then repeated by the second investigator (PC). Without a third investigator any conflicts in coding and analysis had to be resolved to “mutual satisfaction”. As the researchers were a student (AN) and supervisor (PC) it is possible that due to the “power-relationship” within the relationship that this was biased towards the supervisor (PC) of the research group which may have affected the trustworthiness of the analysis.

### Triangulation

Triangulation with quantitative data or other qualitative methods was not practically feasible and therefore not undertaken to enhance the trustworthiness of the data.

## Results

### Participants

Eighteen members of the year-five cohort were eligible for inclusion and volunteered to participate. Saturation was achieved after ten interviews, with no new additions to the code-book. Five male and five female UG students with a mean age of 26-years participated. The mean length of time since attending the symposium was 4 months. Two participants agreed to be interviewed, but withdrew before the interview, in both cases because they had other activities that prevented them from attending the interview. All participants were asked, and responded to all the questions in the interview guide. Interview duration ranged between 18 and 30 min. No interviews were stopped due to political comments about the government or derogatory comments regarding individuals.

### Themes

The analysis was scaffolded on the three themes of SDT, and are described here, namely; autonomy, competence and relatedness. Several other themes were identified during the interviews, namely; i. Satisfaction with peer-teaching, ii. Peer-teaching as a concept, iii. Practical issues related to the peer-teaching session and iv. Teaching style from faculty. Due to the volume of transcript data and because of their previous description in the literature, we have not included the descriptions and results of these themes and rather focus on the concepts found within self-determination theory.

### Theme: autonomy

#### Subtheme – Autonomous learning

The need for autonomy refers to our desire to be our own source of our behaviour [21]. Participants worked in groups, and as groups autonomously identified different methods to prepare for the symposium. Participants also reported to have autonomously discovered additional helpful learning materials and developed their own study habits on an individual basis, without their group:

*“Also, it is like it creates the culture of reading to yourself and be confident enough to deliver a given topic.”* [Interview 2].

Autonomy reflects the experience that behaviour is an expression of the self and generates a complete feeling of free will to choose whatever a person considers or feels right to do [3]. A component of autonomy is that individuals may choose to not engage with the activities, either due to a lack of interest, or other priorities. A number of students reported that they did not prepare for their presentations:

“*They said that they give us slides and we go prepare them and teach each other. For me, I think it has no sense. Giving me slides and then go and teach. I even didn’t prepare them.”* [Interview 3].

In developing the symposium, the faculty chose to give the students an assigned topic and pre-prepared PowerPoint slides. This will have naturally limited the student’s autonomy but was felt to be a necessary balance to ensure the symposium kept to time and that the sessions were factually correct. This decision appears to have been supported by the students as they reported their own reservations about delivering incorrect information.

*“As we are students, you can express yourself in bad ways, and you give bad information to others”* [Interview 9].

#### Subtheme – desire for supervision

Despite developing their own strategies for learning and preparing for the symposium, participants reported a desire for the supervision of their peer-teaching.

*“It would then be better, if we have like two supervisors to complement us, because, yes it is good to teach your colleagues and learn from your colleagues but at our level, we may have something which we don’t understand, I don’t know, and you don’t know as my colleague and this if we are doing it only with medical students, we will all leave the room without having an answer for that question, I emphasize on the supervision of that session, because you may have wrong answer from your colleagues or you don’t get an answer of that question”* [Interview 5]

It is interesting to note that all the participants identified their own strategies to prepare for the session. Therefore, they did not request supervision of this autonomous process of preparation. Instead they described a desire for supervision of the symposium itself, to ensure that the material shared was factually correct.

### Theme: competence

#### Subtheme – enhanced learning of the content

The session was designed so that students would gain the theoretical knowledge needed in order to better provide clinical care and enable them to engage with the non-peer-teaching practical sessions (simulation). Participants consistently reported that the content of the symposium was necessary for their future career as a clinician

*“And most of the time it's part of emergency medicine, and every department has emergency cases and specifically the ABCDE, I think for the neonates or for the patients in paediatrics won’t be the same as in old people, even the patient handling in paediatrics and adults, I think it's a bit different.”* [Interview 6]

Retaining theoretical information is important. Participants reported that they felt that they had a better retention of the information because they had engaged in the teaching process.

*“you more remember it, and it's more retained because you were teaching”* [Interview 5]

*“I think it was an important step from our consultants to try to expose us at least in the presentations, because the more you present the more learn about something, and the more you talk about something, the more you retain it in your mind*” [Interview 6].

#### Subtheme – competence in teaching

Participants described that they felt more competent as teachers and were motivated to be educators in the future, though we have no evidence as to whether this desire was present before the symposium.

*“So, I felt more motivated that I can even be a teacher for other times in the future, maybe I can use other ways of teaching be it bed side teaching, but however it is I will be teaching”* [Interview 4]

Many students had not previously felt competent in teaching. One of the encouraging themes identified was that the students felt that they had developed their competence in teaching and public speaking. Students reported feeling positive that they require teaching skills, as medical educators, not only of healthcare students, but also of their patients.

*“So it has many, two very, very importances, Helping us to understand well the topics and helping us to be teachers of others, teach each other and to speak in the public which is very and very important”* [Interview 1]

*“So, first having such experience of being in front of people and having such experience of talking in front of people, feeling comfortable and getting such time, it was the first thing important for me and as medical students we have to have such skills because, we will be having the tasks of teaching even our patients”* [Interview 6]*.*

#### Subtheme – competence: overcoming anxiety

This was the first time that most students had been asked to teach their peers. For many of the students, this prospect created anxiety and fear. Therefore, one step towards competence was developing confidence and overcoming this anxiety. Students reported the importance of practicing (rehearsing) the presentation in order to perform better, and reduce anxiety.

*“So, you have to try to eliminate such kind of frustration and fear so that you can keep what have in your mind and trying to maintain your audience because the target is to teach the audience and give the required knowledge. So, I had to practice, practice, and practice in order to give the audience”* [Interview 6]

However, despite participants feeling anxious, the anxiety reduced for most students. In addition, participants reported a strong sense of satisfaction when they were able to overcome their fear and deliver a presentation to their peers.

*“And even in the first minutes, you may be threatened when you are standing in front of others, but as the time goes on, you feel comfort and feel how you organize the talk.”* [Interview 7]

*“Yeah, it is good. It was so good to teach my classmates as I felt just free, with no fear and as I have prepared my materials, when I was preparing my materials, I tried to read and go deep so that I came up with good explanations to my colleagues”* [Interview 1]

### Theme 3: relatedness

Relatedness is the desire to feel connected with others, caring and being cared for, and having a sense of belongingness, both with significant other individuals and a significant community [3, 44]. The symposium was designed to enable students to work in groups and therefore optimize this sense of relatedness. Students were positive regarding the group-work nature of the peer-teaching.

*“We prepared in group and we participate. We shared ideas, and we take decisions of what to talk, for sure we shared with them, we discuss what to talk. In other words that work was prepared by the whole group.”* [Interview 7]

*“Yeah, it was really helpful and was really happy to work with them. Because even if we were three [members in the group], I was the first one to present at the time. They focused more on what I was about to say than their tasks. Because even if we were three, we worked more on my part than others parts. It was really helpful to work with them.” [Interview 8]*

Participants valued being able to teach, and contribute to the learning of their peers.

*“I was feeling happy and good because, I helped my classmates and group mates to gain something about what I read.”* [Interview 6].

The anxiety related to teaching, reported by participants, has been described above. Students reported that working with and teaching peers reduced this anxiety because of the relationship they had with each other.

*“And it was easy because these were my colleagues, it was really easy and enjoyable. I wasn’t afraid to ask as they are my colleagues they couldn’t laugh at me if I don’t know something. And even when I was about to be with them, I had no fear. So, it was easy.”* [Interview 10]

Acknowledgement and reward are known to create a tremendous feeling of relatedness, as students are being taken seriously as emerging colleagues [3]. Participants valued the encouragement they received from their peers during the symposium.

*Interviewer “After the time you conducted this session, Did you feel more confident in being a teacher?” Response: “YEAH!!!. EXACTLY. After presenting my topic, my colleagues were very excited and gave me hand clap.”* [Interview 2].

## Discussion

This qualitative study sought to explore and better understand the lived experiences, attitudes and perceptions of medical students as peer-teachers at the University of Rwanda when participating in a Peer-Assisted Learning intervention in the pediatric department. In Rwanda, undergraduate medical teaching activities are classically didactic, with senior clinicians and professors delivering the teaching. Therefore, peer-teaching, as a style of learning was relatively novel to the students.

### Autonomous learning

Activities that support autonomy have been found to have a positive association with motivation [3, 17, 21–25]. An overarching goal of medical education is progressive independence of the learner [45]. This is especially important in settings such as Rwanda, where graduates will often be placed in isolated, rural hospitals and will need to maintain their own learning related to their local population’s health needs. Students reported that they benefited a great deal from their autonomous preparation, group work, and reading in the period before the symposium. Students were not given a summative assessment for the symposium; therefore, there was no summative external motivator. Though no students directly reported that they felt “more motivated”, the preparation activities they described suggest that they were intrinsically motivated to develop themselves, expand their own knowledge, identify learning resources and to deliver a good presentation that was factually correct.

### Autonomy support

Clinical educators and teachers are a vital source of organizational, pedagogic and affective direction for students [20]. Autonomy support is defined as the degree to which instructors acknowledge students’ perspectives and encourage their proactive participation in learning activities [21]. Students felt the need to be actively supervised by the faculty during the symposium. Students are rarely fully autonomous, and they value pedagogic support and rely on teachers to manage and support the learning environment and process [20, 21]. Interestingly, they were able to autonomously develop their own strategies to prepare and deliver the teaching sessions; however, they desired supervision of the delivery of the teaching to ensure that the information shared was factually correct. This desire for support has the potential to inhibit peer-teaching in resource-limited settings where faculty numbers are low. If peer-teaching activities are incorporated routinely into a medical curriculum in settings such as ours, then an important piece of research work would be to explore if students autonomously develop their own processes of self-monitoring and their own systems of peer-feedback within the student group. Just as our students have described a desire and a need for engagement and support to autonomously develop their own teaching skills, it would be interesting to explore if they share the same desire and need to autonomously develop their own peer-feedback skills. Students will only learn from a PAL activity if they attend the activity. Even when activities are mandatory it is possible that having a faculty member present is a powerful extrinsic motivator to ensure attendance and participation. We postulate that self-monitoring by students may result in a reduction in attendance and levels of engagement, with the removal of this extrinsic motivator.

### Competence – learning the materials/content

Peer-teaching improves the acquisition of knowledge and skills [8–10]. Participants reported that they felt that they had improved their understanding and knowledge content of the symposium and that they felt that they had a better retention of the material. Participants reported that this was partly because they had prepared for the sessions and therefore, had felt it necessary to read around the topic. They also felt a sense of responsibility to deliver a presentation that was factually correct. Engaging with learning materials in an active manner, such as those described by our participants, is known to enhance learning, retention and processing of the information [1, 5, 6, 46]. SDT suggests that this increased sense of competence will also result in an increase in the IM of the students.

### Competence of teaching

Within the theory of SDT, the desire for competence is defined as the desire “to feel effective in whatever actions one pursues and performs”, furthermore, “competence is not meant as attained skill or ability per se, but rather a perception of confidence and effectance” [3]. It is well established that PAL offer ‘tomorrow’s doctors’ the opportunity to practice their teaching skills at an early stage in their careers [4, 47]. Our participants reported having improved their confidence in teaching skills, public speaking, their ability to communicate with others and being able to distil complex medical information. This was an encouraging finding as these skills have the potential to not only improve their teaching skills but also to hopefully improve the care that these students are able to provide to patients in their future medical practices. Some medical graduates will go on to develop a specific interest, and career, in medical education, and it was an encouraging finding in our study that the peer-teaching inspired participants towards this. Even for those who did not report wanting to pursue a career in medical education, almost all will be involved, at some point, in the education of their peers during their medical career after graduation. Some may feel that they do not have the competence to do so and therefore may not be intrinsically motivated to this. Therefore improving their sense of competence in this area, during undergraduate studies, is important as they become life-long teachers in their own working environments [15, 16, 48].

### Competence – overcoming fear

The need for competence leads us to seek challenges and to persistently attempt to maintain and enhance capabilities in the working situation. Nervousness or anxiety in certain situations is normal, and public speaking is no exception. Several students autonomously identified repetitive practice as a means to overcome this anxiety. Peer-teaching in other settings has been documented to cause anxiety in the peer-teacher, which was also reported by our participants [12–14]. Though similar to previous studies, our participants reported that this decreased during the preparation. It has also been reported in the literature that as more PAL activities are established in curricula that this learner anxiety reduces and that peer-teaching prepares students for future careers/roles as clinician-teachers and medical educators [12, 14]. The anxiety experienced by students preparing for peer-teaching activities in the peer-teaching model needs to be addressed with adequate supervision, where available. Anxiety should not inhibit these teaching methods, as in the long-term student anxiety will diminish, and they will be more confident and competent educators.

### Relatedness

The responses to the questions of how students felt when they were working in their groups and attending presentations, were positive in that there was a strong sense of connection with other peers which brought a sense of security [15]. This is consistent with other studies that suggest a feeling of safety seems to be connected to students’ perception of increased learning and independence [49]. Problem-based learning activities have also been shown to stimulate relatedness, as groups of students are required to collaboratively work on problems [3]. This concept of relatedness drawn from working collaboratively was also reported by our participants.

Social, economic, ethnic and cultural factors, are important, playing on the extrinsic and intrinsic motivation of students. The relational nature of peer-teaching is important, and, though not explored in this study, may offer a real opportunity, to contribute to other steps that are required to mitigate for some of these socio-cultural factors that are found in Rwanda and other settings. This would make an interesting piece of future exploratory work.

### Transferability (external validity) of findings

We focused on year-five medical students at the University of Rwanda. Being a competent teacher is a skill that should be encouraged in all medical graduates, and activities to cultivate this throughout medical school should be encouraged. Though there are challenges to engaging students with PAL activities, our experience in this setting suggests that students could be encouraged to commence such activities much earlier in their studies. Motivation to enter medical school is often externally driven for Rwandan medical students. Though not measured, our personal experience is that external factors, such as school and parents, play the largest role in motivating Rwanda high-school students to pursue medical studies. This has important implications for their own levels of IM during medical school. Activities that stimulate IM throughout medical school should be encouraged. The findings of this study show that PAL has a real potential to engage students and stimulate autonomy, competence and relatedness and therefore increase intrinsic motivation. With appropriately designed educational activities, peer-teaching could be commenced earlier in the curriculum to not only develop teaching skills but also intrinsically motivate students. The findings are also applicable to other medical schools within the region, many of whom share the same pathologies, communities, resources and teaching styles.

### Strengths

This is the first study to assess PAL in Rwanda. It has used qualitative methods to deeply explore the lived experiences, attitudes and perceptions of these medical students.

### Limitations

Our study was prone to the biases of data analysis that are commonly found in qualitative research, such as reliance on first impressions, a tendency to ignore conflicting information, and emphasizing data that conforms with the underlying theory or biases. This study was limited in that it did not examine the evidence and variables suggesting the long term effects of PAL and peer-teaching on IM [50]. We used partially deductive analysis, based on the pre-determined theory of SDT, and this limits our analysis in a manner which is somewhat inflexible, and potentially limited the in-depth exploration of the data, adding potential biases. Due to the volume of data produced we have been unable to describe several other themes that were identified during the interviews, namely; i. Satisfaction with peer-teaching, ii. Peer-teaching as a concept, iii. Practical issues related to the peer-teaching session and iv. Teaching style from faculty. Finally, an audit trail was not kept during the methodology development, interviews or coding.

### Future research

No assessment was made to quantifiably measure the gain in knowledge or to compare it with other learning strategies. An interesting piece of research work would be to quantitatively assess and compare any correlation between motivation (e.g. with the SMMS questionnaire) and knowledge acquisition (e.g. using Multiple Choice Questions), comparing peer-teaching and other, more standard, teaching methods for the setting.

## Conclusion

It is now accepted that enhancing student autonomy, competence and sense of relatedness will augment the intrinsic motivation (IM) of the student. Peer-Assisted Learning (PAL) has been shown to be equally or more effective then faculty-delivered teaching. It has previously been acknowledged in the literature that careful thought should be given to how PAL is implemented within medical schools and that this implementation will depend on the aim of the peer teaching, whether to reduce pressure on faculty and resources, or whether for the educational development of the peer tutors. The PAL used here demonstrates that in addition to these factors there is the potential to increase IM whilst increasing knowledge acquisition and teaching skills. We postulate that students in resource-limited settings, such as Rwanda, would benefit from regular PAL activities and that in the long term this will not only enhance student learning, competence and experience but potentially change the workload and demands on faculty whilst enhancing intrinsic motivation in students.

## Supplementary information

**Additional file 1.**

**Additional file 2.**

## Data Availability

The datasets generated and/or analysed during the current study are not publicly available due to the requirement for approval from the local ethics committee but are available from the corresponding author on reasonable request and ethical approval.

## Abbreviations

AMEE: Association for Medical Education in Europe
CHUK: University Teaching Hospital of Kigali
CMHS: College of Medicine and Health Sciences
EM: Extrinsic motivation
IM: Intrinsic motivation
PI: Principal investigator
pRRAPID: Pediatric recognition and responding to acute patient illness and deterioration
SDT: Self-determination theory
SMMS: Strength of Motivation for Medical School
UG: Undergraduates
UoL: University of Leeds
UR: University of Rwanda
