# Supplementary material for: Peer-teaching at the University of Rwanda - a qualitative study based on self-determination theory
Source: BMC Med Educ. 2020 Jul 20;20:230. doi: 10.1186/s12909-020-02142-0 (PMC7370529; doi:10.1186/s12909-020-02142-0)
Supplement: Supplementary file 1 — Additional file 1. [file 12909_2020_2142_MOESM1_ESM.docx]

**Supplementary File 1: Study Interview Guide**

- Introductions (about the interviewer and the project)
- Clarify consent
- Any participant questions about the study?
- How did you enjoy your pediatric placement? (Ice-breaker).
- Tell me about the teaching session when you had to teach each other on ABCDE?
- Did you enjoy having to teach your class-mates?
- How long did it take you to prepare for this session? How did you prepare to give your presentation?
- You gave a talk in a group, with other classmates, how many students were in your group? Did you have a chance to practice with the other members of your group?
- What was it like to work with the other students in the group?
- What do you think might be the benefits of us teaching each other?
- What could be some of the problems with sessions like this?
- Do you think we should do more sessions like this in other departments, where we teach other?
- After the session do you feel more confident in being a teacher?
- Do you think that this session has made you more motivated to be a teacher in the future?
