# Supplementary material for: Peer-teaching at the University of Rwanda - a qualitative study based on self-determination theory
Source: BMC Med Educ. 2020 Jul 20;20:230. doi: 10.1186/s12909-020-02142-0 (PMC7370529; doi:10.1186/s12909-020-02142-0)
Supplement: Supplementary file 2 — Additional file 2. [file 12909_2020_2142_MOESM2_ESM.docx]

**Study title: Peer-teaching at the University of Rwanda. A qualitative study based on Self-Determination Theory**

**Supplementary File 2: Coding tree of SDT analysis**

| **Theme - autonomy** |
| --- |
| Student led teaching of sessions |
| Autonomous selection of reading topics |
| Autonomous reading |
| Autonomous teaching |
| Self-reflection |
| **Theme - Competence** |
| In-depth reading |
| Constructive feedback |
| Concept understanding |
| Increased confidence after presenting |
| Searching for other information sources during preparation |
| Desire for competence |
| Confidence in public speaking and teaching |
| Information processing |
| Re-enforcement of learning |
| Relevance to clinical studies |
| Retention of knowledge |
| Leadership skills |
| **Theme - relatedness** |
| Peer-student discussion resulting in learning |
| Size of study/presentation group |
| Team work gave confidence in task |
| Team building |
| Feeling safe when peer is teaching |
| Connection with others |
| Encouragement from faculty after presenting |
| Division of tasks |
